# Resveratrol: a supplementation for men or for mice?

**DOI:** 10.1186/1479-5876-12-158

**Published:** 2014-06-03

**Authors:** Valentina Ponzo, Laura Soldati, Simona Bo

**Affiliations:** 1 Department of Medical Sciences, University of Turin, Turin, Italy; 2 Department of Health Sciences, University of Milan, Milan, Italy

**Keywords:** Polyphenoles, Resveratrol, Wine

## Abstract

Resveratrol is a polyphenolic compound found in several plants. In the last decades, the interest in this compound has enormously increased after benefits on metabolism and increased lifespan of various organisms have been reported with its supplementation. Several in-vitro and animal studies have observed that resveratrol can act on multiple molecular targets, including sirtuins, a class of NAD + -dependent deacetylases. Despite the enthusiastic results reported in many animal- and in-vitro studies, few trials have been performed in humans with contrasting results. These conflicting data may be due at least in part to differences in the characteristics of the patients enrolled, the dosages and the duration of supplementation. Furthermore, many questions remain still unsolved, such as the dose or the duration of treatment to maximize its effects, the bioavailability of resveratrol and the role of food matrix to improve its bioactivity.

In conclusion, at present the use of resveratrol as a supplement is not yet justified by the existing evidence.

## Background

The phytoalexin resveratrol (3,5,4’-trihydroxystilbene), a non-flavonoids polyphenolic compound found in many plants such as grapes, and in red wine was first discovered in 1940 [1,2].

Resveratrol owes its fame to the so-called "French Paradox". In the early 90s, the term derived from the observation that the French adults showed a relatively low incidence of coronary heart disease in comparison to the corresponding-age individuals from other Western countries, despite a high dietary intake of saturated fatty acids. The French scientist Renaud attributed this paradox to the increased consumption of red wine [3] and, later, the high resveratrol content of wine has been identified as one of the potential mechanisms of the benefits of red wine [4]. From that moment, the interest in this natural compound has grown enormously. Research has thereafter shifted from human to animal models and the number of studies on resveratrol has increased extraordinarily since 1997, when its anti-cancer properties have been reported [4]. Several authors have investigated the hypothetical cardioprotective, anti-cancer, anti-inflammatory and antioxidant properties of resveratrol. A long list of beneficial effects and many possible direct or indirect molecular targets and mechanisms of action of resveratrol have been described in animal and in vitro studies. Only recently, research has focused on humans again, and so far a number of human clinical trial have been conducted.

## Main text

A milestone in resveratrol research came in the early 2000s when some authors reported that resveratrol is able to mimic caloric restriction effects and increased yeast, worms, flies and rodents lifespan [5,6]. This effect was ascribed to the resveratrol ability to activate sirtuin proteins, NAD ^+^-dependent de-acetylase involved in the regulation of metabolism, apoptosis, mitochondrial biogenesis, inflammation, fatty acid metabolism, and glucose homeostasis [7-10]. But sirtuins are not the only target of resveratrol and thanks to its ability to act on numerous molecular targets, resveratrol exhibits versatile biological effects: from the inhibition of angiogenesis, cancer cell and metastatic cell growth [11-24] to the improvement of vascular function, arterial blood pressure values, and platelet aggregation [25-27], and the anti-inflammatory [28,29], antioxidant [30,31] and hypoglycaemic effects [32-38].

To date, the human clinical trials available have shown conflicting or controversial results about the anticancer, cardioprotective and anti-inflammatory properties of resveratrol [39-50]. Other sources of controversy derive from the fact that many of the hypothesized mechanisms have not yet been demonstrated in humans [27]. However, some positive results have been found. A recent meta-analysis of 11 human clinical trials, investigating the effects of resveratrol on the metabolic pattern of type 2 diabetic patients, points out a beneficial effect of resveratrol consumption on fasting glucose and glycosylated haemoglobin values, and insulin resistance measured by the homeostatic model assessment [51].

Why have not all the promising results found in preclinical studies been confirmed in humans?

Differences in formulation, dosage of resveratrol and duration of follow-up may at least in part justify these conflicting results. However, many other issues remain unresolved. For example, the dose of resveratrol to maximize its effects without safety concerns is still unknown; similarly the duration of supplementation remains a matter of concern (a high dose for a short-term or a low dose for a long period?). Moreover, the relationship between the low bioavailability and the high bioactivity of resveratrol, the so-called resveratrol paradox, has not yet been solved. Since this molecule is rapidly metabolized and its concentrations quickly disappear in the blood, it is difficult to justify its benefits, while the knowledge of metabolites or mediators responsible for the effects of resveratrol is still scanty [52].

The majority of *trans*-resveratrol metabolites were found to be derived by glucuronidation or sulfation. Since the rapid metabolism seems to be one of the major reasons for the lower effect on humans, a part of the research is focusing on how to improve the bioavailability of resveratrol. One way to improve the bioavailability may be the combination of resveratrol with other agents that inhibit its metabolism in vivo. Studies have shown that natural compounds, including quercetin and piperine (an alkaloid derived from black pepper), can inhibit glucuronidation and sulfation of resveratrol [53-55]. However, further studies are needed to assess whether these combinations may determine an increase in its bioavailability.

The role of resveratrol in an "epigenetic diet", a diet with a potential impact in delaying ageing and preventing age-related diseases, could be a further topic worthy of investigation.

Finally, another interesting but unsolved topic is the possible influence of the food matrix on the bioavailability of resveratrol in humans. Some authors have shown that trans-resveratrol is better absorbed when assumed through wine or grape juice than from tablets [56]; in vivo bioavailability and bioactivity could be increased by the food matrix because of the presence of other natural compounds, such as other polyphenols that seem to play a synergic role and increase resveratrol bioactivity [57].

## Conclusions

The versatile properties of resveratrol found in in-vitro and animal studies have not been unequivocally confirmed in human studies. At present, resveratrol should be assumed by natural foods, mainly through the moderate consumption of red wine, grape juice or peanuts, while the consumption as a supplement is not justify by the existing scientific literature. Many issues remain certainly open, and a resveratrol formulation with increased bioavailability and effective in humans as in mice has yet to be discovered.

## Competing interests

The authors declare that they have no competing interests.

## Authors’ contributions

VP participated in the conception of the manuscript, literature collection and interpretation, manuscript writing and revision. LS participated in the conception of the manuscript, literature interpretation, manuscript writing and revision. SB participated in the conception of the manuscript, literature interpretation, manuscript writing and revision. All authors have read and approved the final manuscript.
